# Engineering Bispecific Peptides for Precision Immunotherapy and Beyond

**DOI:** 10.3390/ijms262010082

**Published:** 2025-10-16

**Authors:** Xumeng Ding, Yi Li

**Affiliations:** Academy of Pharmacy, Xi’an Jiaotong Liverpool University, Suzhou 215123, China; dingxumeng2022@163.com

**Keywords:** self-assembling peptides, peptide drug delivery, dual-targeting strategy, immune checkpoint blockade, precision immunotherapy

## Abstract

Bispecific peptides represent an emerging therapeutic platform in immunotherapy, offering simultaneous engagement of two distinct molecular targets to enhance specificity, functional synergy, and immune modulation. Their compact structure and modular design enable precise interaction with protein–protein interfaces and shallow binding sites that are otherwise difficult to target. This review summarizes current design strategies of bispecific peptides, including fused, linked, and self-assembled architectures, and elucidates their mechanisms in bridging tumor cells with immune effector cells and blocking immune checkpoint pathways. Recent developments highlight their potential applications not only in oncology but also in autoimmune and infectious diseases. Key translational challenges, including proteolytic stability, immunogenicity, delivery barriers, and manufacturing scalability, are discussed, along with emerging peptide engineering and computational design strategies to address these limitations. Bispecific peptides offer a versatile and adaptable platform poised to advance precision immunotherapy and expand therapeutic options across immune-mediated diseases.

## 1. Introduction

**Figure 1:**
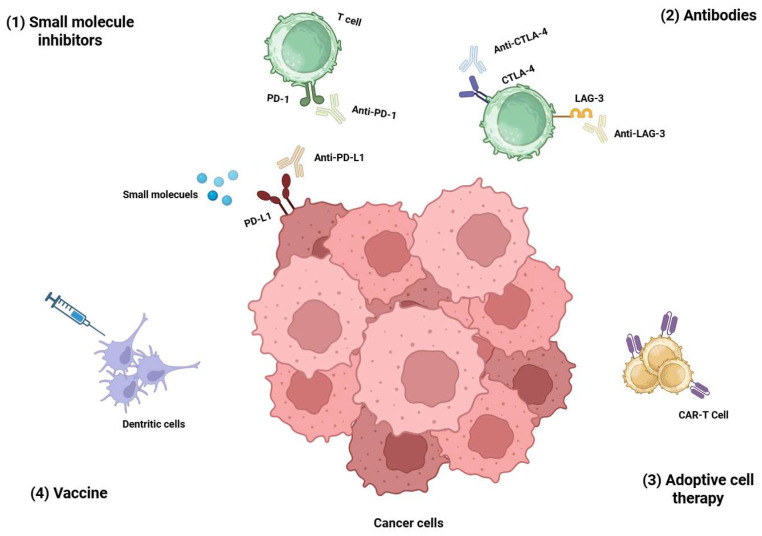
Overview of key immunotherapeutic strategies in cancer treatment. The figure illustrates four primary modalities: (1) Small molecule inhibitors targeting intracellular signaling pathways; (2) Monoclonal antibodies that block surface receptors or immune checkpoints; (3) Adoptive cell therapies, such as CAR-T cells engineered to recognize tumor antigens; and (4) Cancer vaccines that prime T-cell responses through antigen presentation. These approaches highlight the evolution of precision immunotherapy. Created in https://BioRender.com.

Cancer immunotherapy has revolutionized oncology by harnessing the host immune system to destroy malignant cells (Figure 1) [1]. The most notable approaches include immune checkpoint inhibitors (ICIs), such as anti-PD-1 and anti-CTLA-4 antibodies, which have shown significant clinical benefits for various cancers. However, these therapies are often limited by immune-related side effects, tumor-intrinsic resistance, and variable patient responses [2,3]. In parallel, small molecule inhibitors targeting intracellular signaling pathways have also contributed to the immuno-oncology landscape, although they frequently suffer from off-target effects and limited immune specificity [4]. These limitations have fueled interest in alternative modalities that provide greater specificity, better safety profiles, and more flexible mechanisms [5].

Among the emerging strategies in immunotherapy, peptide-based therapeutics offer a compelling solution to many of the shortcomings associated with antibodies and small-molecule inhibitors [6,7]. Their intermediate molecular size enables better tissue penetration than antibodies, while maintaining a higher degree of target specificity than many small molecules [8]. Peptides can be rationally designed to mimic key functional domains involved in immune signaling, allowing them to disrupt protein–protein interactions, modulate receptor activity, or engage immune effector cells with high precision. In addition, their synthetic accessibility, structural flexibility, and amenability to chemical modification make peptides well-suited for rapid development and optimization [9]. And a report shows that the global peptide therapeutics market is experiencing rapid growth, valued at USD 117.26 billion in 2024 and projected to reach USD 260.25 billion by 2030, with a CAGR of 10.77% [10]. Taken together, these features not only underscore the growing significance of peptide-based therapeutics as versatile in immunotherapy but also provide a strong foundation for the development of next-generation modalities such as bispecific peptides.

The concept of bispecific molecules was first introduced in the 1960s by Nisonoff and colleagues, who demonstrated the feasibility of re-associating different antigen-binding fragments, an early precursor to bispecific formats [11]. In the 1980s, this approach laid the foundation for the development of bispecific antibodies, which have since demonstrated notable clinical potential, particularly in redirecting immune cells to tumors and targeting multiple disease pathways concurrently [12]. Rather than simply combining two monospecific agents, bispecific molecules are typically engineered through chemical linkage, enabling synergistic effects that can enhance potency and reduce off-target toxicity [13,14]. Furthermore, these integrated structures often exhibit more predictable pharmacokinetics and lower risks of drug–drug interactions [15]. In contrast, traditional combination therapies may have uncertain mechanisms and less controllable interactions [16]. The clinical validation of this strategy is exemplified by the FDA approval of several bispecific antibodies, such as amivantamab (approved for NSCLC, 2021) and cadonilimab (approved in China, 2022), marking a pivotal advancement in the therapeutic application of bispecific platforms [17].

**Figure 2:**
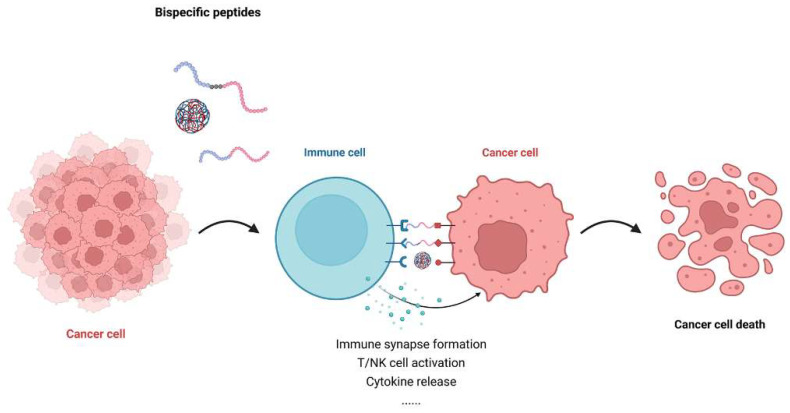
Schematic representation of bispecific peptide-mediated cancer cell killing. Bispecific peptides bridge immune cells with cancer cells by simultaneously engaging immune receptors and tumor-associated antigens. This facilitates immune synapse formation, leading to immune cell activation, potent cytokine release, and subsequent cancer cell death.

Based on this dual-targeting principle, researchers extended the idea to peptides, leveraging their small size, enhanced tissue penetration, and relative ease of chemical synthesis. Bispecific peptides not only hold promise for improving therapeutic efficacy and safety but also provide a strategy to overcome the intrinsic limitation of weak binding affinity through cooperative or multivalent target engagement (Figure 2) [18]. Compared with more established bispecific formats such as antibodies, fusion proteins, or small molecules (Table 1), bispecific peptides offer unique advantages. Their compact structure enables access to shallow or spatially restricted binding sites, many of which are considered “undruggable”, while also ensuring deeper tissue penetration and intracellular targeting [19]. In addition, they generally exhibit lower immunogenicity, improved tissue distribution, and simpler, cost-effective synthesis. Furthermore, bispecific peptides are synthetically accessible, allowing precise control over sequence, conformation, and chemical modifications. Such flexibility permits the incorporation of non-natural amino acids or cyclization to enhance proteolytic stability and in vivo half-life. Collectively, these attributes position bispecific peptides as a versatile therapeutic class capable of achieving both structural precision and pharmacological flexibility [20].

This review provides a comprehensive overview of bispecific peptide design strategies, their mechanistic functions, and the translational challenges they pose. Specific focus is given to architectural formats, including fused, linked, and self-assembled constructs, and their roles in mediating immune synapse formation, dual checkpoint blockade, and tumor microenvironment (TME) remodeling. Emerging solutions for stability, delivery, immunogenicity, and manufacturing scalability are also discussed. Collectively, these advances underscore the growing relevance of bispecific peptides as a modular platform in the development of next-generation precision immunotherapies.

## 2. Current Design Strategies of Bispecific Peptides

**Figure 3:**
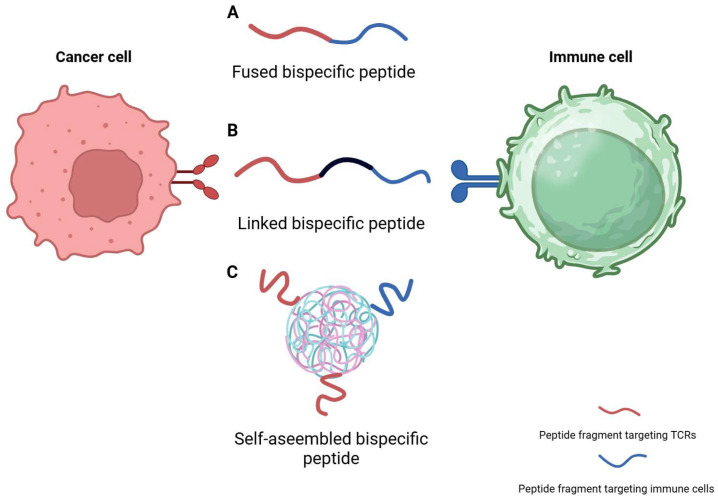
Structural classifications of bispecific peptides in immunotherapy. (A) Fused bispecific peptide composed of two functional domains linked in a single chain. (B) Linked bispecific peptide formed by covalently connecting two peptide fragments with a flexible linker. And (C) Self-assembled bispecific peptide, where modular peptide units spontaneously oligomerize into multivalent complexes for enhanced immune targeting. Created in https://BioRender.com.

A systematic framework for categorizing bispecific peptides provides critical insights into how structural variations shape their pharmacological and immunotherapeutic properties. Based on the spatial configuration and integration strategy of their dual-targeting domains, these molecules can be broadly divided into three principal formats: fused, linked, and self-assembled (Figure 3). This classification reflects fundamental differences in chemical assembly and molecular architecture, which in turn influence critical attributes such as target-binding affinity, tissue penetration, proteolytic stability, and manufacturability. Organizing bispecific peptides by assembly mode enables comparative evaluation of their mechanisms and informs rational design strategies, setting the stage for optimizing efficacy, specificity, and translational potential in next-generation peptide-based immunotherapies.

### 2.1. Fused Bispecific Peptide

Fused bispecific peptides consist of single, continuous polypeptide chains that inherently recognize two distinct targets, often without the need for additional linkers. Many of these peptides were originally designed to bind a single receptor or epitope, but subsequent structural or functional analyses revealed a secondary binding capability. Their simple architecture confers advantages such as streamlined manufacturing, lower molecular weight, and predictable pharmacokinetics, making them appealing candidates for further optimization and therapeutic development, even when their dual-targeting properties arise serendipitously.

**Figure 4:**
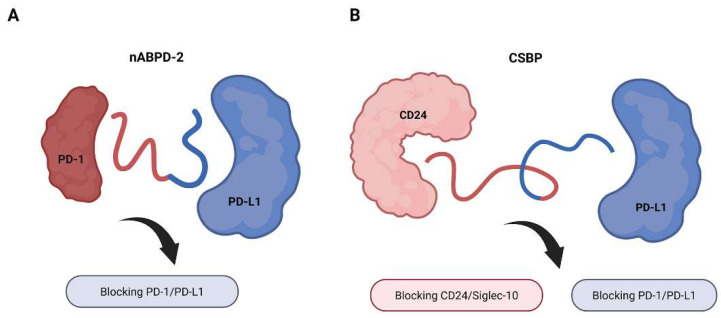
Representative fused bispecific peptides targeting immune checkpoints and tumor markers (black arrows). (**A**) The peptide nABPD-2 simultaneously blocks PD-1 and PD-L1, enhancing T-cell-mediated cytotoxicity in tongue squamous cell carcinoma. The red line is the peptide fragment targeting to PD-1 and the blue line is the other one targeting PD-L1. (**B**) The CSBP peptide dual-targets CD24 and PD-L1, disrupting the CD24/Siglec-10 and PD-1/PD-L1 axes, and reprograms myeloid cells to inhibit tumor growth, particularly in synergy with radiotherapy. The red line is the peptide fragment targeting to CD24 and the blue line is the other one targeting PD-L1. Created in https://BioRender.com.

A prime example of fused bispecific peptide design is the PD-1/PD-L1-targeting nABPD series developed by Wang et al. The series exemplifies a rational structure-guided approach to bispecific peptide development, beginning with the moderate-affinity PD-1 binder nABPD-1 (KD = 11.9 nM) [27,28]. The nABPD-2 variant was engineered to optimize binding, as sequence alignment revealed 38.5% homology with the PD-L1 binding interface, and computational docking identified critical interactions (ARG21-GLU61, TRP22-PHE63, and HIS37-TYR68) (Figure 4A). This Y-shaped peptide maintains structural flexibility while incorporating both PD-1 and PD-L1 binding motifs, achieving balanced high-affinity interactions (KD = 5.21 nM for PD-1 and 4.74 nM for PD-L1). Functional characterization demonstrated sustained target engagement (>6 h) and enhanced T-cell activation, with 2.1-fold greater IL-2 secretion compared to monospecific antibodies. The compact 5 kDa structure offers better tumor penetration while avoiding antibody-related issues such as Fc effector functions and vascular exclusion, along with the manufacturing benefits of solid-phase synthesis [8]. This study highlights how iterative structure–function optimization can transform a modest peptide binder into a clinically relevant bispecific inhibitor with defined pharmacological advantages.

Building on the theme of rational, structure-guided optimization, a subsequent study by Shen et al. further exemplifies the profound impact of strategic peptide engineering on stability and function [29]. Their work began with the identification of a linear L-amino acid peptide (BP) that blocked the CD24/Siglec-10 interaction. Recognizing the inherent susceptibility of L-peptides to proteolytic degradation, they implemented a sophisticated D-amino acid substitution strategy to confer hydrolysis resistance. Crucially, alanine scanning was first employed to identify key residues (L3, F4, V5) critical for CD24 binding, which were preserved in their native L-form to maintain activity. The remaining residues were substituted with their D-configuration counterparts, and a retro-inverse peptide (PBP-1) was also designed. Hydrophilic glutamic acid modifications were introduced at the peptide termini to address solubility. This rational design process culminated in the creation of CSBP (Figure 4B), a stabilized D-peptide that exhibited superior serum stability, resisting degradation for over 96 h while retaining its high-affinity blocking activity. The most profound finding was that CSBP functioned as a novel dual-targeting therapeutic, capable of concurrently inhibiting both the CD24/Siglec-10 and PD-1/PD-L1 immune checkpoint pathways. This bispecific functionality enabled CSBP to synergize with radiotherapy, significantly enhancing the phagocytosis of tumor cells by macrophages and monocytic myeloid-derived suppressor cells (M-MDSCs), while simultaneously activating CD8^+^ T cells, thereby engaging both innate and adaptive immune mechanisms to suppress tumor growth in both responsive and resistant models.

### 2.2. Linked Bispecific Peptide

In contrast to fused bispecific peptides integrating two functional domains into a single continuous polypeptide chain, linked bispecific peptides adopt a more modular architecture by connecting separate peptide segments via a covalent linker. The linked design offers greater flexibility in choosing independently optimized peptide domains and adjusting their spatial orientation. The use of customizable linkers, ranging from flexible Gly-Ser (GS) repeats to rigid α-helical segments, allows for the fine-tuning of interdomain distance, conformational dynamics, and pharmacokinetic properties [30,31]. Such architectural versatility enables linked bispecific peptides to preserve the functional independence of each domain while facilitating cooperative binding or signaling effects, particularly in cases where steric hindrance or conformational constraints would limit the use of fused formats.

**Figure 5:**
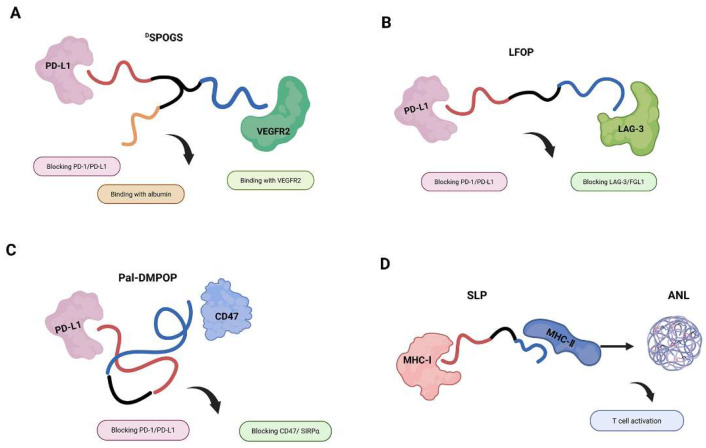
Design strategies for linked bispecific peptides and their immunomodulatory functions (black arrows). (**A**) DSPOGS is a dual-targeting peptide that links PD-L1 (red line) and VEGFR2 (blue line) antagonists via a GS linker (black line), thereby enhancing T-cell infiltration. With the third peptide (yellow line) combining with albumin, ^D^SPOGS achieved longer half-life. (**B**) LFOP combines (black line) a PD-L1-binding peptide (red line) with an LAG-3 blocker (blue line) to disrupt dual checkpoint pathways. (**C**) Pal-DMPOP integrates (black line) PD-L1 (red line) and CD47 (blue line) blockade into a palmitoylated construct, improving tumor macrophage recruitment. (**D**) ANL is a dual-epitope SLP-based vaccine loaded into aluminum nanoparticles to co-activate CD4^+^ and CD8^+^ T-cell responses through MHC-I and MHC-II presentation. The red line is the peptide fragment targeting to MHC-I and the blue line is the other one targeting MHC-II. The black line indicates the linker. Created in https://BioRender.com.

In linked architectures, such as those utilizing non-cleavable linkers, the domains are covalently connected, maintaining functional independence while preventing premature degradation. A notable example is the chimeric peptide ^D^SPOGS, which incorporates two functionally distinct domains OPBP-1 (8–12), a PD-L1-binding peptide, and ^D^A7R, a VEGFR2 antagonist (Figure 5A) [32]. The inseparable GS linker was chosen to preserve the dual-targeting functionality without enzymatic cleavage, ensuring simultaneous blockade of PD-1/PD-L1 and VEGF/VEGFR2 pathways. To enhance pharmacokinetics, OGS was further modified by coupling with ^D^SP, a retro-inverse albumin-binding peptide, via a hydrophilic AEEA-AEEA spacer to minimize steric hindrance. This yielded ^D^SPOGS exhibited high affinity to both human and mouse serum albumin and retained binding to PD-L1. Stability assays confirmed that ^D^SPOGS resisted proteolysis in serum (>96 h) and achieved a 9-fold longer half-life (256 min) in vivo compared to OGS, addressing the rapid clearance typically seen in small peptides. Functional validation demonstrated ^D^SPOGS effectively blocked PD-1/PD-L1 interaction (IC50 ≈ 100 µM) and inhibited HUVEC cell migration/tube formation, confirming dual-pathway inhibition without direct cytotoxicity to tumor cells. This work represents a significant leap in peptide-based cancer therapy, showcasing how rational design and combinatorial strategies can amplify therapeutic outcomes. By bridging innate (angiogenesis inhibition) and adaptive (immune checkpoint blockade) immunity, ^D^SPOGS + radiotherapy sets a new standard for multimodal cancer treatment, with broad implications for future drug development and clinical trials.

The modularity of the linked design strategy is further exemplified by Qian et al. to create LFOP, a conjugate linking the hydrolysis-resistant LAG-3/FGL1 blocking peptide LFP-D1 with the established PD-L1-binding motif OPBP-1 (8–12) (Figure 5B) [33]. LFP-D1 can interact with LAG-3 and effectively inhibit the LAG-3/FGL1 interaction, which represents a novel target for immune checkpoint therapy [34]. This study highlights the significant potential of peptide-based immune checkpoint inhibitors, particularly the novel LAG-3/FGL1 blocking peptide LFP-D1 and the LFOP, in advancing cancer immunotherapy. By specifically targeting non-redundant inhibitory pathways (LAG-3/FGL1 and PD-1/PD-L1), these peptides enhance T-cell activation and tumor infiltration, offering advantages such as lower immunogenicity, improved tumor penetration, and cost-effectiveness compared to antibodies. Furthermore, the synergistic effect of LFOP with radiotherapy underscores a promising strategy to overcome immunosuppression in the tumor microenvironment, paving the way for more effective and safer combination therapies in clinical oncology.

Another study conducted by Hu et al. also demonstrated the principle of covalent linkage for sustained dual blockade. The chimeric peptide Pal-DMPOP was engineered through a rational design strategy that merges structural optimization and chemical modification to achieve dual blockade of CD47/SIRPα and PD-1/PD-L1 pathways (Figure 5C). Its DMP domain derives from the CD47-targeting peptide pep-20 (2–10), optimized via molecular dynamics simulations to introduce key mutations (M3/W6) that enhance binding affinity to CD47, while the OPBP-1 (8–12) domain ensures protease resistance [35]. To further improve pharmacokinetics, a palmitic acid tail was appended to the N-terminus, leveraging albumin-binding for prolonged circulation, a tactic validated in clinical peptides like liraglutide [36]. This modular architecture not only addresses stability challenges (e.g., enzymatic degradation) but also enables synergistic immune activation by spatially coordinating dual checkpoint blockade, as demonstrated by its superior efficacy compared to equimolar peptide mixtures. The design emphasizes practical principles for peptide-based therapeutics, including computational refinement of interactions (e.g., using ZDOCK/MOE), strategic incorporation of non-natural elements (such as D-amino acids and fatty acid tags), and deliberate domain linkage to optimize targeting (e.g., lymph node accumulation) and bioactivity.

In the development of peptide vaccines, short peptides often present limitations, including a short half-life due to peptidase degradation and suboptimal efficacy in targeting MHC-I epitopes [37,38]. The study by Bai et al. demonstrates the potential of aluminum nanoparticles (ANLs) to enhance peptide vaccine efficacy through rational design and delivery optimization. The authors engineered a dual-epitope synthetic long peptide (SLP) by linking MHC-I (SIINFEKL) and MHC-II epitopes (ISQAVHAAHAEINEAGR) via a protease-cleavable double-Arg (RR) linker, ensuring simultaneous activation of CD8^+^ and CD4^+^ T cells [39]. To address the instability of peptides, the SLP was modified with five aspartic acids (D) at the N-terminus, enhancing electrostatic adsorption to aluminum nanoparticles. The ANLs were stabilized using a PEG derivative (PpASE), which improved lymphatic drainage due to their uniform size (~100 nm) and negative surface charge [40] (Figure 5D). Notably, ANLs exhibited superior antigen presentation in dendritic cells (DCs), with 2-fold higher MHC-I epitope display compared to free peptides and induced robust T cell proliferation in vitro. In vivo, ANLs elicited potent cytotoxic T lymphocyte (CTL) responses (~77% specific lysis) and synergized with anti-PD-1 therapy, achieving complete tumor regression in 25% of melanoma-bearing mice. The study underscores the importance of modular peptide design and nanocarrier optimization in overcoming limitations of traditional peptide vaccines. These findings provide a blueprint for developing next-generation nanovaccines with clinical translatability, particularly for personalized cancer immunotherapy.

The selection of an appropriate linker is a critical determinant of the structural and functional integrity of linked bispecific peptides. Linkers govern the spatial orientation, conformational flexibility, and steric separation between the two targeting domains, directly impacting dual-target engagement and biological activity [41,42]. Commonly employed linkers include polyethylene glycol (PEG), peptide-based sequences, and cleavable motifs responsive to environmental stimuli.

PEG linkers, due to their hydrophilicity and flexibility, are frequently incorporated to enhance solubility, reduce renal clearance, and prolong systemic circulation. The chain length of PEG affects both pharmacokinetics and the spatial reach between binding moieties [43,44,45]. Peptide-based linkers offer the advantages of biodegradability, biocompatibility, and modular design. Their composition and length can be precisely controlled during solid-phase peptide synthesis. Flexible linkers often comprise small, uncharged residues such as glycine and serine, which minimize steric hindrance and allow conformational freedom between domains [46]. Conversely, more rigid or structured peptide linkers (e.g., α-helical linkers rich in alanine or leucine) may be employed to enforce specific spatial orientations [47]. The use of immunologically inert or protease-resistant sequences can further improve in vivo stability [48]. Cleavable linkers are designed to undergo site-specific degradation in response to physiological cues, such as pH changes, reducing environments, or enzymatic activity. Disulfide-based linkers are responsive to intracellular reductive conditions and are often utilized to enable selective activation or release of functional domains inside cells [49]. Enzyme-sensitive linkers, such as those cleavable by tumor-associated proteases (e.g., matrix metalloproteinases, cathepsins), allow for extracellular activation in the tumor microenvironment. Such linkers enable a degree of spatiotemporal control, improving the therapeutic index and minimizing systemic toxicity.

The choice of conjugation chemistry must align with the linker characteristics to preserve bioactivity and synthetic feasibility. For PEG linkers, maleimide–thiol coupling is commonly used to link cysteine-containing peptides to PEG-maleimide derivatives, forming stable thioether bonds. Alternatively, NHS ester–amine conjugation allows attachment through lysine residues but may require protection strategies to avoid nonspecific reactions [50]. Peptide linkers are often synthesized directly during solid-phase peptide synthesis (SPPS), allowing full control over sequence and length. When assembling two peptide fragments post-synthesis, native chemical ligation (NCL) or enzymatic ligation (e.g., sortase A-mediated) can provide site-specific covalent linkage without requiring harsh conditions. For cleavable linkers such as disulfide bridges, oxidative coupling of cysteine residues enables reversible dimerization of peptide units [51]. Alternatively, click chemistry, particularly strain-promoted azide–alkyne cycloaddition (SPAAC), offers a bioorthogonal and efficient route for linking peptides bearing azide or alkyne handles, and is compatible with both PEG and peptide-based linkers. Collectively, the integration of rational linker design with compatible chemical conjugation strategies provides a modular and adaptable framework for constructing linked bispecific peptides with optimized stability, pharmacokinetics, and target engagement.

### 2.3. Self-Assembled Bispecific Peptides

Taking modularity a step further, self-assembled bispecific peptides harness the power of spontaneous molecular organization to create multivalent structures, unlocking new opportunities for enhanced binding avidity, cooperative signaling, and innovative immunotherapeutic applications [52]. In contrast to structurally rigid bispecific constructs, these peptides are designed to undergo dynamic oligomerization in situ, triggered by external cues such as receptor binding, enzymatic activation, or physicochemical changes in the tumor microenvironment (e.g., pH or redox gradients) [53,54]. This self-assembly process leads to the formation of higher-order nanostructures or multivalent complexes, which can significantly amplify local peptide concentration, facilitate receptor clustering, and improve target avidity. The resulting constructs often exhibit enhanced immune synapse formation and more potent cytotoxic activity, while maintaining the synthetic tractability and modularity inherent to peptide-based platforms.

**Figure 6:**
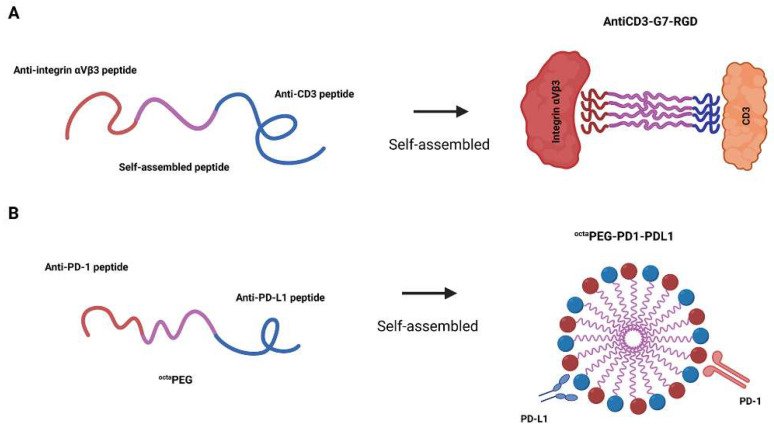
Self-assembling bispecific peptide constructs for targeted immune activation. (**A**) TRA peptide integrates anti-CD3 and anti-integrin αvβ3 domains via a G7 self-assembly motif. Upon receptor binding, the construct oligomerizes to facilitate T-cell engagement and tumor cytolysis. (**B**) ^octa^PEG-PD1-PDL1 is a bispecific peptide–polymer conjugate that anchors PD-1- and PD-L1-binding peptides to an eight-armed PEG scaffold, enhancing immune synapse formation and therapeutic accumulation in tumors. Created in https://BioRender.com.

The bispecific peptide antiCD3-G7-RGD exemplifies a rationally engineered construct that combines two distinct targeting motifs: antiCD3 (AKMGEGGWGANDY) for CD3 engagement and RGD for integrin αvβ3 binding, linked through the self-assembling G7 (GNNQONY) core (Figure 6A) [55]. This architecture leverages orthogonal targeting domains positioned at opposite termini to maintain independent receptor binding capabilities. And the central G7 module serves as both a structural spacer and an inducible assembly switch, undergoing β-sheet fibrillization only upon receptor-mediated stabilization [56]. Controlled valency of self-assembling core amplifies receptor clustering beyond simple bivalent binding. Importantly, the G7 spacer’s length (~2.1 nm) was optimized to permit simultaneous engagement of CD3 on T cells and integrins on tumor cells without steric hindrance, as confirmed by TEM and SPR studies. Specifically, the localized assembly of G7 at immune synapses generates sustained mechanical forces that amplify T-cell receptor (TCR) signaling, as demonstrated by a 3.2-fold increase in IFN-γ production compared to monovalent controls. The system is modular and adaptable to a broad range of cell-pairing applications because either targeting motif can be substituted without disrupting the core assembly mechanism.

Extending the concept of self-assembly to engineered multivalent scaffolds, Zhang et al. developed a bispecific peptide–polymer conjugate. The bispecific ^octa^PEG-PD1-PDL1 combines PD1pep (SNTSESF) and PDL1pep (NYSKPTDRQYHF) motifs on an 8-arm PEG scaffold, leveraging multivalency and spatial organization to enhance immune synapse formation (Figure 6B) [57]. Key to its design is the precise 1:1 stoichiometry of peptide conjugation, achieved through maleimide-thiol chemistry and confirmed by ^1^H-NMR, which ensures balanced targeting of PD-1 on T cells and PD-L1 on tumor cells. The branched architecture of the PEG core optimizes steric accessibility, enabling simultaneous receptor engagement while avoiding interference, which is a critical advantage over linear bispecific constructs. This spatial control, coupled with multivalent avidity, drives efficient T cell–tumor bridging (60% conjugation vs. 25% controls) and prolonged circulation (t1/2(β) = 34 h). The design’s modularity allows substitution of alternative targeting peptides, provided they incorporate cysteine for site-specific coupling and maintain sub-micromolar affinity to compensate for monovalent weakness. By integrating polymer-enhanced clustering with peptide specificity, this platform overcomes the traditional limitations of bispecific peptides, such as rapid clearance and low avidity, while enabling mechanical reinforcement of immune synapses to potentiate cytotoxicity.

Self-assembled bispecific peptides represent a versatile and forward-looking design paradigm that capitalizes on non-covalent interactions or environmental triggers to induce the formation of multivalent architectures. This dynamic assembly enhances functional avidity and promotes cooperative binding to multiple receptors, thereby improving target specificity, immune cell engagement, and cellular uptake. The inherent reversibility and modularity of self-assembly not only facilitate fine-tuning of structural and functional properties but also support the incorporation of additional delivery-enhancing features, such as controlled release mechanisms or tumor-selective activation [55]. Moreover, the ability to engineer responsive assembly in situ allows for spatially confined therapeutic activity, potentially minimizing off-target effects and improving pharmacokinetic behavior. These attributes position self-assembled bispecific peptides as a promising frontier in the design of next-generation immunotherapeutics.

## 3. Mechanism of Action of Bispecific Peptides in Immunotherapy

Bispecific peptides exert their therapeutic effects through dual-targeting mechanisms, enabling precise immune modulation, magnifying antitumor cytotoxicity, and remodeling the TME (Table 2). By simultaneously engaging distinct molecular targets, these constructs orchestrate coordinated activation of immune effector cells and blockade of immunosuppressive pathways, thereby overcoming key resistance mechanisms associated with single-agent immunotherapies.

### 3.1. Immune Synapse Formation and T Cell Engagement

One of the principal mechanisms of bispecific peptides is to physically link immune effector cells to tumor cells, forming artificial immunological synapses that drive targeted cytotoxic responses. In this configuration, bispecific peptides contain distinct binding domains that engage receptors on both immune effector cells and tumor-associated antigens expressed on malignant cells. This proximity-driven interaction promotes receptor clustering and crosslinking on immune cells, initiating intracellular signaling cascades that lead to effector activation, cytokine secretion, and cytolytic activity [61,62].

**Figure 7:**
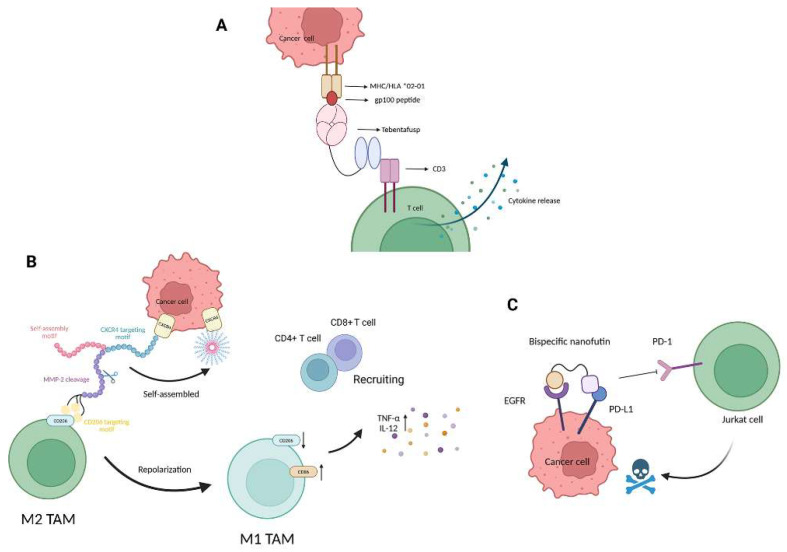
Mechanisms of action for bispecific peptides in immune cell redirection and tumor targeting. (**A**) Tebentafusp bridges gp100-positive tumor cells and CD3^+^ T cells, redirecting T-cell cytotoxicity and enhancing local IFN-γ production. (**B**) bsGP targets CD206 and CXCR4, reprogramming TAMs to an inflammatory M1 phenotype and facilitating T-cell infiltration. (**C**) B10-B11, a bispecific Nanofitin, conditionally blocks PD-L1 in EGFR-overexpressing tumors, ensuring tumor-restricted immune activation and minimizing off-target effects. Created in https://BioRender.com.

Metastatic uveal melanoma (mUM) is a highly aggressive malignancy with a poor prognosis and currently lacks effective standard therapies. Tebentafusp, an innovative bispecific protein, represents a novel therapeutic approach [58] (Figure 7A). Through its TCR domain, the molecule binds with high specificity to the melanoma-associated peptide gp100. This surface antigen is highly expressed in melanocytes and melanoma cells and simultaneously engages CD3 on T cells via an anti-CD3 single-chain variable fragment (scFv). This dual-targeting mechanism creates a physical link between tumor cells and T cells, thereby enabling the formation of immune synapses and the activation of polyclonal T cells, regardless of their native TCR specificity, to mediate potent tumor cell lysis [63]. At the molecular level, treatment with tebentafusp has been shown to significantly upregulate IFN-γ pathway-related markers, particularly inducing the release of chemokines such as CXCL10, which promotes the recruitment of CXCR3, expressing effector memory T cells into the TME [64].

Beyond T cells, bispecific peptides targeting alternative effector mechanisms can engage macrophages or suppressive myeloid populations to remodel the TME. An et al. (2023) developed a novel bispecific glycopeptide (bsGP) that significantly suppresses postoperative recurrence of bladder cancer by simultaneously targeting CD206 on tumor-associated macrophages (TAMs) and CXCR4 on tumor cells, aiming to disrupt the pro-tumorigenic interactions within the TME and prevent tumor relapse after surgery (Figure 7B) [59]. The bsGP reprograms pro-tumoral M2-type TAMs into anti-tumoral M1-type macrophages, thereby enhancing their cytotoxic function and promoting T cell recruitment. Additionally, in the TME, bsGP is specifically cleaved by matrix metalloproteinase-2 (MMP-2), resulting in the release of a CXCR4-targeting fragment that self-assembles into nanofibers. These nanofibers inhibit the CXCR4 signaling pathway, reducing tumor metastasis and improving T cell infiltration. Through its dual-targeting activity, bsGP reshapes the immune landscape of the TME and dramatically reduces the postoperative recurrence rate to 22%, in stark contrast to the much higher recurrence rates observed with standard clinical treatments such as BCG (78%) and doxycycline (89%). This strategy enables precise spatial and temporal control over therapeutic targets, offering a promising direction for future cancer immunotherapy research.

In addition, certain bispecific peptides employ conditional targeting to further refine tumor selectivity and minimize off-target cytotoxicity. For example, the bispecific Nanofitins B10-B11 selectively recognizes tumor cells co-expressing high levels of EGFR and PD-L1 [60] (Figure 7C). B10-B11 selectively binds to tumor cells that co-express high levels of both EGFR and PD-L1, as demonstrated in MDA-MB-231 and MNNG-HOS cells. The observations highlight that B10-B11 activity depends on the simultaneous presence of both target antigens. Moreover, its PD-L1 inhibitory function requires prior engagement with EGFR. Even in cells with high PD-L1 expression, B10-B11 cannot effectively block the PD-1/PD-L1 interaction if EGFR is absent. Consistently, co-culture assays showed that B10-B11 enhances T cell cytotoxicity against dual-positive tumor cells by disrupting PD-L1 signaling, whereas monovalent B11 or controls lacking co-expression of EGFR failed to produce this effect. This dual recognition not only restores T-cell cytotoxicity in EGFR/PD-L1 double-positive tumors but also minimizes immune activation in healthy tissues, offering an additional layer of safety and precision [60].

### 3.2. Checkpoint Inhibition and Costimulatory Reprogramming

In addition to facilitating direct cytotoxicity, bispecific peptides provide a unique platform for the concurrent blockade of multiple immune checkpoint pathways, thereby restoring effector T-cell functionality and reversing exhaustion. Tumors frequently exploit multiple inhibitory axes, such as PD-1/PD-L1 and CTLA-4, to evade immune clearance [65]. Simultaneous inhibition of these pathways via bispecific constructs potentiates immune activation and prevents compensatory resistance mechanisms [66].

An illustrative example of a rationally designed bispecific peptide is nABPD-2, which simultaneously targets PD-1 and PD-L1 to enhance antitumor immunity. This peptide was developed through sequence optimization and truncation. Mechanistically, nABPD-2 acts by competitively binding to both PD-1 on T cells and PD-L1 on tumor cells, thereby disrupting the immunosuppressive PD-1/PD-L1 axis [28]. This dual blockade relieves T cell exhaustion and facilitates the reactivation of antitumor cytotoxic responses. Compared to single-target monoclonal antibodies, nABPD-2 exhibits superior cellular binding stability and longer retention on T cells, with binding sustained up to 6 h, as confirmed by flow cytometry and confocal microscopy. Functionally, nABPD-2 enhances T cell-mediated cytotoxicity against multiple tumor cell lines, including TSCC (CAL27 and CTSC-2), osteosarcoma (MNNG/HOS), and melanoma (A375), outperforming either anti-PD-1 or anti-PD-L1 monoclonal antibodies used alone [67]. Importantly, the peptide does not induce direct tumor cell toxicity but rather potentiates T cell activation, as evidenced by increased IL-2 secretion and mRNA expression in co-culture assays.

Further optimization is achieved through conjugation to multivalent scaffolds. A peptide–polymer conjugate, ^octa^PEG-PD1-PDL1, was developed to simultaneously target PD-1 on T cells and PD-L1 on tumor cells [57]. ^octa^PEG-PD1-PDL1 competitively inhibits the PD-1/PD-L1 interaction, thereby restoring T cell activation and effector function. By co-engaging receptors on both T cells and cancer cells, the conjugate physically bridges these cell types, facilitating immune synapse formation. This bridging effect enhances CTL contact with tumor cells and promotes targeted killing, as confirmed by increased Annexin V staining, IFN-γ secretion, and tumor cell apoptosis in co-culture assays. In vivo studies further confirmed that ^octa^PEG-PD1-PDL1 improves tumor-specific accumulation and reshapes the immune microenvironment by increasing CD8^+^ T cell infiltration and reducing T cell exhaustion markers. These immunomodulatory effects resulted in substantial tumor growth suppression in murine CT26 models, with a tumor inhibition rate of nearly 90%. This approach highlights the therapeutic promise of multivalent, bispecific platforms that integrate immune checkpoint blockade with spatial coordination of target-effector cell interactions.

### 3.3. Mechanistic Enhancement of Antigen Presentation

**Figure 8:**
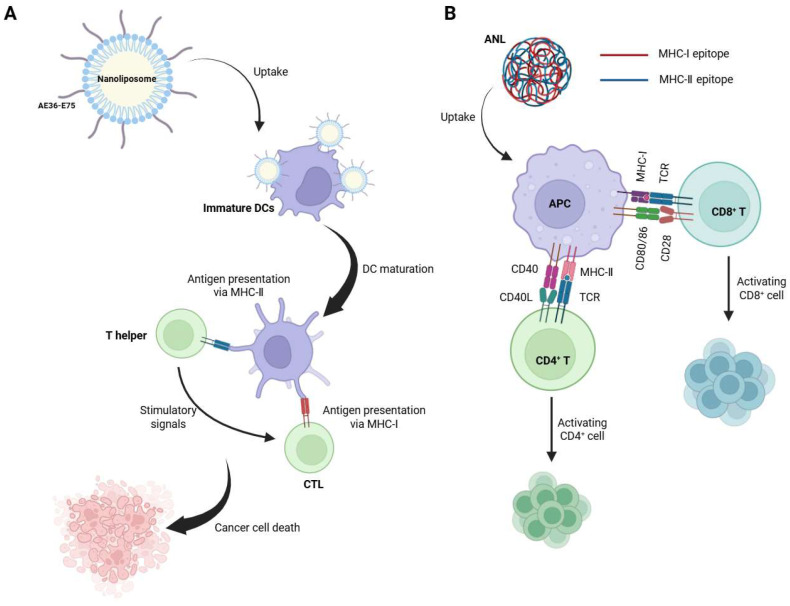
Mechanistic enhancement of antigen presentation by bispecific peptide-based systems. (**A**) Nanoliposomes carrying linked MHC-I (E75) and MHC-II (AE36) epitopes promote dendritic cell uptake, dual antigen presentation, and T cell activation, leading to tumor cell killing. (**B**) Antigen nanoliposomes (ANLs) encapsulating cleavable synthetic peptides deliver MHC-I/II epitopes to APCs, enabling simultaneous activation of CD4^+^ and CD8^+^ T cells via coordinated costimulation. Created in https://BioRender.com.

Bispecific peptides also contribute to robust adaptive immune priming by enhancing antigen presentation. The study by Zamani et al. [38] provides a compelling demonstration of how bispecific peptide design can significantly enhance antigen presentation in cancer immunotherapy. By chemically conjugating an MHC-I epitope E75 and an MHC-II epitope AE36 with an optimized spacer, the researchers created a synthetic long peptide that required professional antigen-presenting cell (APC) processing [38]. This design showed that the bispecific approach forces antigen processing in dendritic cells (Figure 8A). This effect was amplified through DOPE-containing liposomes that protected peptides and promoted endosomal escape, along with PADRE to provide universal T-cell help [68,69]. The nanoliposome induced 2.1-fold higher IFN-γ production versus mixed peptides, enhanced T-cell infiltration, and achieved 105% tumor growth delay. This example serves as an excellent illustration of how molecular engineering can be employed to mimic natural antigen processing pathways. By comparing two distinct approaches, a physical mixture of two short peptides versus a covalently linked bispecific construct, the clear comparative data provides direct experimental evidence supporting the advantages of bispecific designs.

Another study introduces ANLs designed to deliver SLPs containing MHC-I and MHC-II epitopes, linked by a protease-cleavable double-arginine (RR) motif [39]. These nanoparticles are stabilized by a PEG derivative (PpASE) and modified with negatively charged aspartic acids (D) to improve adsorption onto the nanoparticles, achieving high encapsulation efficiency (~95%). The small size of ANLs facilitates efficient drainage to lymph nodes, where they are internalized by APCs, particularly DCs (Figure 8B). Once inside APCs, the RR linker is cleaved by intracellular proteases, releasing the MHC-I and MHC-II epitopes for simultaneous presentation. This dual presentation activates CD8^+^ and CD4^+^ T cells, with CD4^+^ T cells providing critical help to CD8^+^ T cells through cytokine secretion (e.g., IFN-γ, IL-2) and CD40-CD40L interactions, amplifying the CTL response. The ANLs also function as adjuvants, promoting DC maturation (upregulated CD40/CD86) and enhancing antigen cross-presentation. When combined with TLR9 agonist CpG ODN1826, the immune response is further strengthened. In murine tumor models, ANLs have been shown to significantly inhibit tumor growth and prolong survival, demonstrating efficacy comparable to that of protein-based vaccines. Additionally, ANLs effectively activate human DCs, highlighting their potential for clinical translation.

## 4. Emerging Application Beyond Oncology

**Figure 9:**
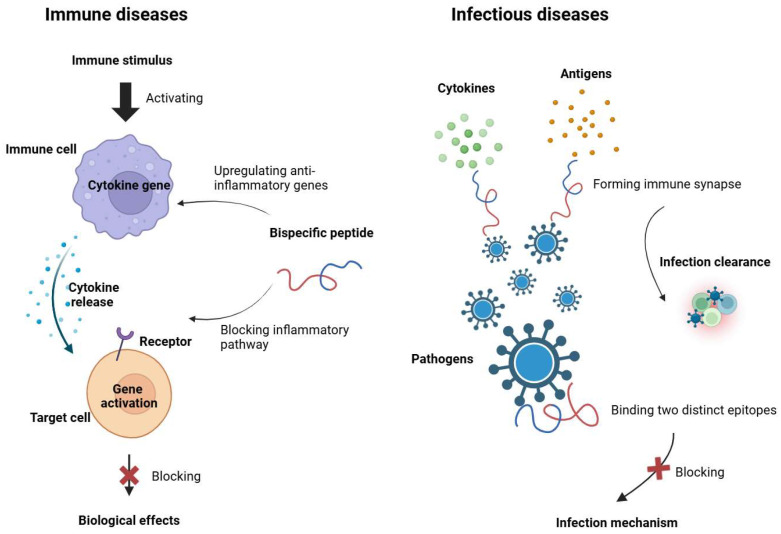
Applications of bispecific peptides in autoimmune and inflammatory diseases and infectious diseases. This figure illustrates the therapeutic mechanisms of bispecific peptides beyond oncology. Left (Immune Diseases): Bispecific peptides can bind cytokines or their receptors to block pro-inflammatory signaling or upregulate anti-inflammatory gene expression, thereby suppressing pathological immune activation. Right (Infectious Diseases): Bispecific peptides facilitate immune synapse formation by bridging pathogen antigens and immune effectors, or by binding multiple epitopes to block pathogen entry and promote infection clearance. Created in https://BioRender.com.

While most bispecific peptide development has focused on cancer immunotherapy, the underlying design principles, including dual-target engagement, modularity, and immune modulation, are equally applicable to a broader range of immune-related disorders. The ability to simultaneously influence multiple immune pathways makes bispecific peptides uniquely suited for complex diseases such as autoimmunity and chronic infections, where dysregulated immune networks and antigenic diversity often undermine single-target interventions (Figure 9). However, extending their application beyond oncology requires tailored strategies that address disease-specific immune mechanisms, antigen expression patterns, and tolerogenic environments.

### 4.1. Autoimmune and Inflammatory Diseases

Autoimmune and inflammatory diseases present a distinct immunological landscape characterized by inappropriate immune activation against self-antigens, chronic cytokine release, and disrupted regulatory networks. In this context, bispecific peptides can serve not merely as antagonists but as immune rebalancers, capable of selectively modulating inflammatory and regulatory pathways in tandem.

One promising direction involves dual blockade of pro-inflammatory cytokines or costimulatory signals. For instance, a bispecific peptide designed to simultaneously inhibit TNF-α and IL-6 signaling could suppress synergistic inflammatory cascades implicated in diseases such as rheumatoid arthritis and inflammatory bowel disease [70,71]. Alternatively, bispecifics could be engineered to block IL-17A while engaging TNF receptors to inhibit effector T cell activity and promote regulatory T cell differentiation, an approach with implications for restoring immune tolerance in autoimmune settings [72,73].

Furthermore, bispecific peptides may enable precise intervention at the interface between immune cells and cytokines. For example, a construct that binds both autoreactive T cells (via CD28 or CTLA-4) and pro-inflammatory cytokines could allow spatially restricted immunosuppression, minimizing systemic effects [74]. Such strategies could outperform broad-spectrum immunosuppressants by maintaining protective immunity while selectively attenuating autoreactivity.

Another potential application is in tissue-specific autoimmunity, such as type 1 diabetes or multiple sclerosis, where bispecific peptides could be engineered to target organ-specific antigens (e.g., insulin or myelin basic protein) and immunomodulatory receptors. This would enable localized immune regulation, reducing collateral damage to healthy tissues. Importantly, the small size and synthetic flexibility of peptides make it feasible to design constructs that accommodate the HLA diversity and immune microenvironment unique to each autoimmune condition.

From a translational perspective, the challenges include identifying disease-specific dual targets, avoiding global immunosuppression, and maintaining peptide stability in chronically inflamed tissues. The success of bispecific antibodies, such as ABT-122 (anti-TNF/IL-17A), in clinical trials supports the concept; however, peptide-based alternatives offer superior tissue penetration and simpler manufacturing. These advantages could be harnessed to develop precision immunomodulators tailored for specific autoimmune phenotypes.

### 4.2. Infectious Diseases

The treatment of infectious diseases, particularly chronic or latent infections, requires immune interventions that are both pathogen-specific and immune-activating, yet balanced to avoid immunopathology. Bispecific peptides are uniquely positioned to fulfill this role through dual-targeted immune redirection, mimicking mechanisms observed in natural antiviral immunity and engineered biologics.

In viral infections such as HIV or hepatitis B, where immune exhaustion and antigenic escape are prevalent, bispecific peptides could be engineered to simultaneously bind viral envelope proteins and costimulatory receptors (e.g., CD137, CD28) on T cells or NK cells. This would enable immune redirection toward infected cells, enhance cytotoxic responses, and potentially overcome viral immune evasion strategies [75]. Such constructs could also be paired with latency-reversing agents to reactivate and then eliminate viral reservoirs, a concept currently being tested with bispecific antibodies [76].

In the context of bacterial infections, bispecific peptides have the potential to simultaneously engage pattern recognition receptors (PRRs) and bacterial toxins or surface antigens, thereby promoting pathogen clearance while modulating inflammatory responses. For example, targeting both TLR2 and lipoteichoic acid might activate innate immunity while controlling excessive cytokine release in sepsis [77].

In pandemic preparedness, bispecific peptide platforms may enable rapid generation of dual-epitope constructs targeting conserved viral regions, facilitating the design of multivalent vaccines or T cell-activating immunotherapies for future outbreaks. Moreover, combining bispecific peptides with innate immune agonists or antiviral peptides could synergistically enhance both immune activation and pathogen clearance.

## 5. Challenges and Limitations

While bispecific peptides offer significant promise as next-generation immunotherapeutics, their clinical translation remains constrained by a range of challenges. Key limitations include physicochemical instability, susceptibility to enzymatic degradation, potential immunogenicity, suboptimal delivery and bioavailability, and complexities in large-scale manufacturing. Addressing these issues is essential for realizing the full therapeutic potential of bispecific peptide platforms in oncology and beyond.

### 5.1. Stability and Degradation Concerns

One of the foremost challenges facing bispecific peptides is their limited stability in physiological environments. Due to their small size and flexible conformation, linear peptides are highly susceptible to rapid enzymatic degradation by proteases present in blood and tissues. This instability significantly reduces their half-life, leading to diminished therapeutic efficacy and necessitating frequent administration [78,79]. For instance, studies have shown that unmodified linear peptides often exhibit half-lives of less than 30 min in human plasma, rendering them unsuitable for systemic administration without modification [78]. The challenge becomes even more complex for bispecific peptides, where degradation of either targeting domain can result in the loss of specificity, compromising not only binding affinity but also the therapeutic mechanism. Furthermore, bispecific peptides are prone to chemical instability, such as oxidation of Met, Cys, and His residues or deamidation of asparagine, especially under storage or formulation conditions. These chemical alterations can affect peptide conformation, aggregation behavior, and biological function.

While various stabilization strategies have been proposed, such as cyclization [80], backbone modifications [81], or incorporation of D-amino acids [82], each introduces trade-offs in terms of synthesis complexity, cost, and potential immunogenicity. For example, cyclization may improve proteolytic resistance but could also hinder conformational flexibility required for dual target engagement [80]. And research indicates that while D-amino acid modifications improve stability, they may lead to the generation of non-natural conformational epitopes, thereby increasing the risk of immunogenicity [83]. Thus, the issue of stability remains a significant barrier to clinical translation, particularly for bispecific constructs that require prolonged circulation or tumor localization.

### 5.2. Immunogenicity and Unintended Immune Response

Although peptides are generally considered to be less immunogenic than antibodies, bispecific peptides are not immuno-silent. Their synthetic or engineered nature, especially when incorporating non-native sequences or chemical modifications, can introduce immunogenic epitopes that are recognized by the host immune system, leading to both humoral and cellular immune responses.

The bispecific peptides, with higher molecular weights and more complex structures, involve multiple chemical modifications, such as PEGylation and cyclization, leading to risks of introducing new binding fragments to proteins and altering the initial interaction of peptides with the immune surveillance mechanism. A review points out that non-natural sequences PEGylation may generate new epitopes, disrupting immune tolerance [84]. In addition, there would be a high possibility of forming aggregation and misfolding peptides during the synthesis, formulation or storage, which can be recognized as danger signals that cause activated APCs to increase immunogenicity [85]. These factors call for the need for careful structural design and rigorous quality control to minimize unintended immune activation and ensure the safety of bispecific peptide therapeutics.

### 5.3. Delivery Challenges and Bioavailability Issues

A common limitation in peptide drug development is their inherently short half-life, typically ranging from a few minutes to several hours [86]. In contrast, modified analogs such as liraglutide exhibit significantly prolonged half-lives, reaching up to 11–16 h [87]. As fundamental biological molecules, peptides are highly susceptible to enzymatic degradation; peptide bonds can be cleaved by proteolytic enzymes (e.g., protein hydrolases) before the therapeutic agent reaches the tumor site, thereby compromising efficacy [37,88].

The hydrophilic nature of peptides, along with their polar or charged residues, limits their ability to permeate the lipid-rich cellular membrane, resulting in poor oral bioavailability [89]. Moreover, efflux transporters, cellular defense mechanisms that expel xenobiotics, may recognize therapeutic peptides as foreign substances, further reducing oral absorption and hindering tumor penetration [90].

Bispecific peptides, often engineered for high binding affinity to tumor-associated antigens, may become sequestered at the tumor surface rather than penetrating deeper into the tumor mass. This phenomenon, referred to as the “binding site barrier,” significantly limits their distribution in tumors, particularly in tumors with high antigen expression [91]. Furthermore, despite being smaller than monoclonal antibodies, bispecific peptides still face challenges crossing the abnormally structured and selectively permeable tumor vasculature, which further restricts their effective accumulation within tumor tissues.

### 5.4. Manufacturing and Scalability Issues

In addition to biochemical challenges, the large-scale production of bispecific peptides remains a significant and multifaceted hurdle that must be addressed to enable their widespread clinical application. Although peptides are generally more straightforward to synthesize than full-length proteins or antibodies, the complexity inherent in bispecific peptide design poses substantial manufacturing challenges. The inclusion of sophisticated structural elements, such as complex chemical linkers that join two distinct binding domains, the incorporation of non-natural or D-amino acids to enhance stability, or cyclization to improve conformational rigidity, introduces additional steps and increases the risk of synthesis inefficiencies. These modifications often require specialized reagents and optimized protocols, which can complicate solid-phase peptide synthesis (SPPS) workflows and extend production times [92].

Moreover, as bispecific peptides often involve longer sequences or multi-domain architectures, achieving consistently high purity and yield becomes increasingly difficult. The longer the peptide chain, the greater the likelihood of incomplete coupling reactions, side reactions, or aggregation, all of which contribute to batch-to-batch variability [93]. This variability not only affects the overall yield but can also impact the therapeutic efficacy and safety profiles of the final product. Post-synthetic modifications, such as conjugation of fluorophores, PEGylation, or glycosylation, add further layers of complexity, necessitating rigorous purification and characterization steps to ensure product homogeneity [94].

From a regulatory standpoint, the manufacturing challenges are compounded by the absence of standardized guidelines specific to bispecific peptides. Unlike monoclonal antibodies or small molecules, regulatory frameworks for these novel therapeutics remain under development, creating uncertainty that can prolong approval timelines and increase development costs. Manufacturers must often work closely with regulatory agencies to establish critical quality attributes, validation methods, and stability criteria, all of which require extensive documentation and testing [95].

## 6. Future Perspectives and Emerging Trends

### 6.1. AI and Computational Peptide Design

The increasing role of computational approaches in the development of small molecules has also extended to the design of peptide-based therapeutics. In contrast to small molecules, which require a specific binding pocket to interact with target proteins, peptides do not rely on such structural constraints for binding. However, the flexibility of peptides and the complexity of peptide–protein interactions present significant challenges in predicting binding patterns, highlighting the crucial role of artificial intelligence (AI) and machine learning in peptide drug design [96]. To address common limitations associated with peptide therapeutics, such as rapid degradation, poor metabolic stability, and short systemic half-life, various chemical modifications are employed to enhance their pharmacokinetic and pharmacodynamic profiles. Computational approaches play a pivotal role in these efforts, enabling the prediction of binding conformations, identification of critical residues for target engagement, and rational design of polished analogs.

The use of multi-omics data, such as proteomics, genomics, and transcriptomics, has become an essential strategy for discovering novel peptide candidates for bispecific therapies. By analyzing genomic data that highlight tumor-specific mutations, proteomic data that identify overexpressed or unique protein targets, and transcriptomic data that reveal immune-related gene activity, researchers can pinpoint peptides that target both tumor-associated antigens and immune checkpoint pathways [97]. This integrated approach accelerates the process of discovering bispecific peptides and also allows for the creation of personalized therapies based on the unique tumor characteristics and immune profiles of individual patients.

These approaches integrate structure-based modeling, multi-omics data, and machine learning to guide the optimization of bispecific peptides, aligning with current trends in drug discovery pipelines.

### 6.2. Novel Conjugation Strategies and Synergistic Approaches

Many peptide-based molecules targeting clinically validated proteins have already demonstrated therapeutic value in clinical settings, laying a solid foundation for future innovation. Building upon these validated scaffolds, researchers are now exploring combination strategies that increase specificity, potency, and functional versatility. These creative approaches, particularly in bispecific peptide conjugation, hold considerable promise for advancing the precision and efficacy of immunotherapy.

One promising direction involves the incorporation of oncolytic peptides, which can exert direct tumor-lytic effects while simultaneously stimulating antitumor immunity. Several optimized variants, such as LTX-315, have entered clinical trials, highlighting their translational potential [98]. Beyond conventional dual-target recognition, the fusion of mechanistically distinct peptide motifs represents a promising strategy to reprogram intracellular trafficking and enhance bioactivity. A compelling example is the hybrid oncolytic peptide NTP-385, which was engineered by covalently conjugating the mitochondria-targeting peptide LTX-315 with rhodamine B [99]. While the parent peptide LTX-315 primarily acts through membranolysis and mitochondrial disruption, its hybrid counterpart was rerouted to the nucleus. This subcellular redistribution facilitated the accumulation of reactive oxygen species (ROS) within the nucleus, leading to DNA double-strand breaks and the activation of the intrinsic apoptotic pathway in adherent cancer cells, a population typically resistant to LTX-315. Recent studies also report conjugates between oncolytic peptides and chemotherapeutics, demonstrating synergistic anticancer efficacy [100]. Integration of such cytolytic-immunostimulatory modules into bispecific peptide frameworks could provide a dual advantage of tumor debulking and immune priming, making them attractive candidates for next-generation cancer immunotherapy.

Combining bispecific peptides with antibody frameworks to create peptibodies is another emerging trend, which combines the advantages of both peptides and monoclonal antibodies [101]. By attaching peptides like GE (targeting HER-1) and MY (targeting HER-2) to the Fc region of human IgG1, researchers developed peptibody constructs that can target multiple tumor-associated antigens while also activating immune effector cells. Notably, this approach boosts NK cell-mediated cytotoxicity, showing the potential of peptibodies to enhance immune response and antitumor effects.

Concurrently, bispecific peptides have been incorporated into chimeric antigen receptor T (CAR-T) cell platforms, offering a novel strategy to improve tumor selectivity and reduce off-target toxicity. Traditional CAR-T cells utilize scFvs, which can lead to unintended targeting of healthy tissues. By replacing scFvs with low-affinity targeting peptides, such as L1 (specific for EGFR) and CLT1 (targeting tumor stroma), researchers have engineered bispecific peptide-based CAR-T cells that distinguish cancer cells and normal tissues more precisely [102]. These modified CAR-T cells demonstrated enhanced tumor recognition, deeper stromal penetration, and increased in vivo antitumor activity. Importantly, this strategy led to reduced systemic toxicity and prolonged T-cell persistence, overcoming key limitations of conventional CAR-T therapies.

Together, these advanced conjugation strategies underscore the versatility and transformative potential of bispecific peptides in immunotherapy. Whether integrated into antibody structures, engineered into CAR-T cells, or used as targeting moieties in PDCs, bispecific peptides offer a modular platform for enhancing immune targeting, improving therapeutic efficacy, and minimizing off-target effects. As the field continues to evolve, such innovations are expected to play a pivotal role in shaping the next generation of cancer immunotherapies and beyond.

## 7. Conclusions

Bispecific peptides represent an adaptable and mechanistically precise platform within modern pharmaceutical sciences, combining modular design, immune modulation, and advanced delivery strategies to address complex immunological diseases. Their capacity to engage two molecular targets simultaneously allows for synergistic modulation of immune responses, effectively bridging the gap between immune effector cells and tumor cells, while concurrently neutralizing immune suppressive pathways. This dual functionality not only enhances antitumor efficacy but also holds great potential for tackling autoimmune and infectious diseases, where multifactorial immune dysregulation often undermines the efficacy of conventional monotherapies.

Compared to traditional antibody-based therapies, bispecific peptides offer several intrinsic advantages, including smaller molecular size, improved tissue penetration, synthetic accessibility, and reduced immunogenicity. These features allow for more efficient drug design, rapid iteration, and greater flexibility in addressing emerging therapeutic targets, particularly those previously considered “undruggable,” such as shallow PPI interfaces. Furthermore, advances in peptide engineering, such as cyclization, D-amino acid incorporation, PEGylation, and self-assembly, have significantly developed in vivo stability, bioavailability, and pharmacokinetic profiles.

However, despite the considerable progress in bispecific peptide development, several challenges remain to be addressed. These include rapid proteolytic degradation, limited systemic half-life, potential immunogenicity due to synthetic modifications, and delivery constraints associated with tissue specificity and intracellular access. Addressing these obstacles requires a multidisciplinary approach that integrates rational peptide design, nanotechnology, and systems biology. Computational tools, artificial intelligence, and omics-based screening strategies are increasingly being employed to optimize peptide sequences, predict immunogenic hotspots, and tailor therapeutics to individual patient profiles [102].

Prospectively, emerging platforms such as peptibody conjugates and bispecific peptide-based CAR-T constructs have the potential to reshape current strategies in precision oncology and immune modulation. As these innovations advance from preclinical development to clinical application, bispecific peptides are poised to play a central role in the future of personalized medicine, offering new avenues for the treatment of cancer and immune-mediated diseases.

## Figures and Tables

**Table 1:** Bispecific molecule classes and their distinctive characteristics.

Class	Subtypes/Examples	Features	Representative References
Antibodies	BiTEs, DARTs, CrossMab, KiH	Protein-based, varied half-life and effector function	[21]
Peptides	Cyclic, linear, self-assembling	Small, modifiable, access to shallow epitopes	[8]
Fusion Proteins	Cytokine fusions, decoys	Modular, tunable	[22]
Small Molecules	PROTACs, molecular glues	Orally available, intracellular access	[4]
Aptamers	Dual-aptamer, aptamer-antibody	Nucleic acid-based, reversible binding	[23,24]
Cellular Platforms	CAR-T, TRUCKs	Live-cell therapeutics with bispecific features	[25,26]

**Table 2:** Summary of the immunotherapeutic bispecific peptides developed in recent years.

Class	Compound	Target	Mechanisms of Action	Reference
Immune Synapse Formation and T Cell Engagement	CSBP	CD24 × PD-L1	Blocks CD24/Siglec-10 and PD-1/PD-L1 axes; activates macrophages and CD8^+^ T cells	[29]
	LFOP	LAG-3 × PD-L1	Dual checkpoint blockade enhances T-cell proliferation and IFN-γ production	[33]
	Pal-DMPOP	CD47 × PD-L1	Mobilizes T cells and macrophages; amplifies antitumor immunity via dual signaling	[35]
	antiCD3-G7-RGD	CD3 × αVβ3	Induces CD3 oligomerization and T-cell-mediated cytolysis via self-assembly	[55]
	Tebentafusp	CD3 × gp100	Redirects T cells to gp100^+^ tumor cells; increases IFN-γ and CXCL10 in TME	[58]
	bsGP	CD206 × CXCR4	Reprograms M2 macrophages to M1 phenotype; recruits CD8^+^ T cells	[59]
Checkpoint Inhibition and Costimulatory Reprogramming	nABPD-2	PD-1 × PD-L1	Simultaneously blocks PD-1 and PD-L1; enhances cytotoxic T-cell function	[28]
	^D^SPOGS	VEGFR × PD-L1	Dual targeting facilitates CD8^+^ T-cell infiltration and IFN-γ secretion	[32]
	^octa^PEG-PD1-PDL1	PD-1 × PD-L1	PEG-based scaffold bridges tumor cells and T cells and boosts immune synapse formation	[57]
	B10-B11	EGFR × PD-L1	Conditionally activates PDL1 blockade while minimizing off-target immune toxicity	[60]
Mechanistic Enhancement of Antigen Presentation	Nanoliposome	MHC-I × MHC-II	Increased tumor infiltration of both CD8+ and CD4+ T cells	[38]
	ANL	MHC-I × MHC-II	promotes antigen presentation and activates CD8^+^ T-cell responses	[39]

## Data Availability

No new data were created or analyzed in this study. Data sharing is not applicable to this article.

## Abbreviations

The following abbreviations are used in this manuscript:

APC	Antigen-presenting cell
ANL	Aluminum nanoparticle-loaded long peptide
CAR-T	Chimeric antigen receptor T cell
CTL	Cytotoxic T lymphocyte
CTLA-4	Cytotoxic T-lymphocyte-associated protein 4
CXCR4	C-X-C chemokine receptor type 4
DC	Dendritic cells
EGFR	Epidermal growth factor receptor
Fc	Fragment crystallizable region
FGL1	Fibrinogen-like protein 1
HER2	Human epidermal growth factor receptor 2
IFN-γ	Interferon-gamma
IL-2	Interleukin-2
ICB	Immune checkpoint blocker
LAG-3	Lymphocyte-activation gene 3
MDSC	Myeloid-derived suppressor cell
MHC	Major histocompatibility complex
NK	Natural killer
PD-1	Programmed death-1
PD-L1	Programmed death-ligand 1
PEG	Polyethylene glycol
PPI	Protein–protein interaction
PRR	Pattern recognition receptors
ROS	Reactive oxygen species
scFv	Single-chain variable fragment
SLP	Synthetic long peptide
SPPS	Solid-phase peptide synthesis
TAM	Tumor-associated macrophage
TCR	T-cell receptor
TME	Tumor microenvironment
TNF-α	Tumor necrosis factor alpha
TSCC	Tongue squamous cell carcinoma
VEGFR2	Vascular endothelial growth factor receptor 2
